# Accuracy of Non-contrast-enhanced Quiescent Interval Single-Shot (QISS) Lower Extremity MRA for the Diagnosis of Peripheral Artery Disease: Comparison with CTA and Digital Subtraction Angiography

**DOI:** 10.1186/1532-429X-18-S1-P225

**Published:** 2016-01-27

**Authors:** Akos Varga-Szemes, Giuseppe Muscogiuri, Julian L Wichmann, Paola M Cannao, Stefanie Mangold, Pal Suranyi, Sheldon E Litwin, Carlo N De Cecco, Stephen R Fuller, Shivraman Giri, U Joseph Schoepf, Thomas M Todoran

**Affiliations:** 1 Division of Cardiovascluar Imaging, Department of Radiology and Radiological Science, Medical University of South Carolina, Charleston, SC USA; 2 Department of Medical-Surgical Sciences and Translational Medicine, University of Rome "Sapienza", Rome, Italy; 3 Department of Diagnostic and Interventional Radiology, University Hospital Frankfurt, Frankfurt, Germany; 4 Division of Cardiology, Department of Medicine, Medical University of South Carolina, Charleston, SC USA; 5 Department of Diagnostic and Interventional Radiology, Eberhard-Karls University Tuebingen, Tuebingen, Germany; 6 Scuola di Specializzazione in Radiodiagnostica, University of Milan, Milan, Italy; 7 Siemens Healthcare, Chicago, IL USA

## Background

Quiescent-interval single-shot (QISS) MRA has been recently introduced as a robust non-enhanced MRA technique with image quality reported to be similar to contrast enhanced MRA. The purpose of our study was to evaluate the diagnostic accuracy of QISS MRA in patients with peripheral artery disease (PAD) against invasive digital subtraction angiography (DSA) as reference standard and compare the results to those obtained with CTA.

## Methods

Twenty patients (67 ± 6 years, 11 male) with PAD referred for a clinically indicated lower extremity CTA (SOMATOM Force, Siemens AG, Forchheim, Germany) within 50 days prior to DSA (Axiom Artis, Siemens), were prospectively enrolled and consented for a same-day lower extremity non-contrast MRA on a 1.5T scanner (MAGNETOM Avanto, Siemens AG, Erlangen, Germany) using an investigational prototype QISS sequence (FOV 400 × 260 mm^2^, TR/TE 3.5/1.4 ms, flip angle 90°, acquisition length 144 mm, slice thickness 3.0 mm, in-plane spatial resolution 1.0 × 1.0 mm^2^, and quiescent interval 226 ms). The entire abdominal aorta and lower extremity run-off were covered by eight to nine groups of 48 slices. Image evaluation was performed on a per-segment basis according to an 18-segment model (proximal aorta (1), distal aorta (2), right and left (R+L) common iliac artery (3+4), R+L common femoral artery (5+6), R+L superficial femoral artery (7+8), R+L popliteal artery (9+10), R+L anterior tibial artery (11+12), R+L tibio-peroneal trunk (13+14), R+L posterior tibial artery (15+16), and R+L peroneal artery (17+18). Stenoses were rated on a five-point scale (1-normal; 2-mild (25-50%); 3-moderate (51-75%); 4-severe (76-99%); and 5-total occlusion) by two readers. Sensitivity and specificity were calculated using the McNemar test, while interreader agreement was analyzed with κ statistics.

## Results

A total of 360 segments were evaluated. Thirty-one segments (8.6%) were excluded from the analysis due to image artifacts caused by heavy calcification, insufficient opacification and metal implants. For the remaining 329 segments, moderate stenosis, severe stenosis, and total occlusion were diagnosed by DSA in 23 (6.9%), 36 (10.9%), and 33 (10.0%) segments, respectively. The sensitivity and specificity of QISS MRA in the detection of significant stenosis (>50%) were 84.1% and 92.1% (*P*=NS), respectively. CTA showed a sensitivity and specificity of 89.4% and 94.6% (*P*=NS), respectively. Interreader agreement was excellent for the detection of stenosis by QISS MRA (κ>0.81).

## Conclusions

In this study, diagnostic accuracy of non-contrast QISS MRA was not statistically different from the standard of reference DSA technique, and was comparable to that of CTA. Because non-invasive anatomic evaluation of PAD is essential for treatment planning prior to revascularization, thus non-contrast QISS MRA may provide clinical advantages over contrast-enhanced approaches as this patient population often suffers from renal disease which is associated with complications related to contrast administration.

**Figure 1:**
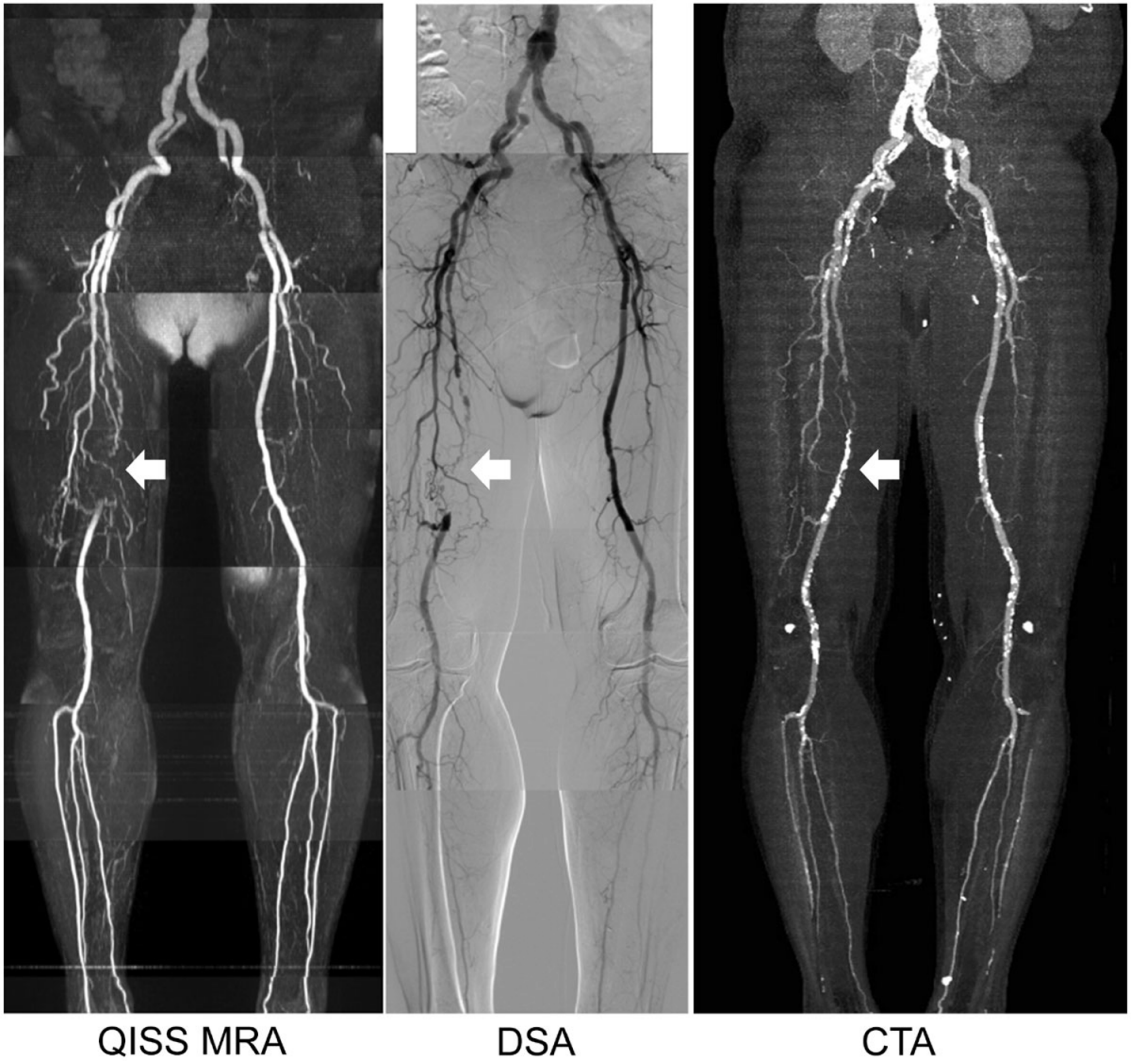
**Representative QISS MRA, DSA and CTA images in a 75-year-old man with right femoral superficial artery occlusion (arrows)**.

